# Fibro-Cystic Uterine Tumor

**Published:** 1880-02

**Authors:** William D. Granger


### FIBRO-CYSTIC UTERINE TUMOR; REMOVAL OF TUMOR AND UTERUS. — BY PROF. JAMES P. WHITE, M. D.

REPORTED BY WILLIAM D. GRANGER, M. D.

Mrs. B., forty-five years old, was in rather feeble health; the tumor had a known history of about a year, and well filled the abdomen, the parietes being tightly stretched over the tumor. It was fluctuating and dull on percussion over the front surface, but tympanitic over the sides. The operation was performed October 6th, 1879. When placed upon the operating table, none of the physicians present doubted the existence of a cystic ovarian tumor. The usual exploratory incision was made, and the tumor exposed. Doubts now arising as to the character of the tumor, a trocar was introduced; no fluid except blood escaped. The trocar being withdrawn, a rapid hemorrhage followed, which was controlled by digital pressure on the wound; the incision was enlarged from the symphysis to above the umbilicus; the tumor was adherent by its upper and right sides; these adhesions were broken down by the operator and his assistant. At one time, when three hands were between the tumor, and the diaphragm, breaking down adhesions, respiration suddenly ceased for half a minute or more; when their hands were removed, respiration was assumed; the heart, however, continued regularly to pulsate; the tumor was covered by a growth of the peritoneal tissue of the uterus, which was opened, and the tumor easily stripped from its covering. It was found to be attached, without a pedicle, to the posterior surface of the uterus. It was removed from its attachment by enucleation. A profuse hemorrhage followed, which was stopped by compressing the abdominal aorta; the hemorrhage coming from the torn surface as from a sponge, and not from any vessels which could be ligated, it was necessary to remove the uterus. Strong ligatures were applied around the cervix, and the uterus removed by the knife. With the uterus, was removed of course the ovaries and the serous sack of the tumor. The wound was closed by deep stitches, and a drainage tube inserted. With and following the last hemorrhage the pulse rose to 160-170, and was weak and fluttering. Subcutaneous injections of ʒii of whiskey, and ʒi of ether in mx. xv doses, noticeably reduced and strengthened the pulse. The patient did not rally well after the operation. The stomach retained all food and medicine given, and there was never the slightest nausea, although so large a quantity of ether was administered. Whiskey, milk punch and beef tea were freely given; every four hours whiskey ℥ii, beef tea ℥ii, quinine gr. v were given by the rectum. At 4 P. M. the pulse was 130, temperature 98½; the day following the operation at 9 A. M., pulse 150, temperature 98½; during the day the pulse rose until it could not be counted, and the patient died thirty-two hours after the operation. This case is interesting, because illustrating a case of abdominal tumor, when the error of diagnosis could not have been foreseen. The removal of the tumor was justifiable, after its nature was known, because of its rapid growth and the correspondent failure of the health, so that the patient said her life was a burden to her. Death in this case is clearly to be attributed to that much abused cause, shock. It plainly was not due to inflammation, or any of the secondary results of inflammation.

Hemorrhage, which was perhaps an assisting element, was a minor one. Although a large quantity of alcohol was given—perhaps by rectum and stomach as much or more than ℥xvi—it had almost no effect upon the pulse, or to rally the patient. And, although the patient was a weak woman and unaccustomed to stimulants, her mind, which was remarkably clear until her death, was unaffected by this large amount. It is an interesting question; what became of it? what force did it exert in the economy? The tumor weighed eighteen pounds. After removal it still gave distinctly a sense of fluctuation, and was of a jelly-like consistency. It was fibro-cystic in structure. The cysts were from a very small size to that of an egg; in places the tumor was honey-combed with small cysts.

